# Effect of the cysteine prodrug L-2-oxothiazolidine-4-carboxylic acid on *in vivo* platelet activation, oxidative stress, and procoagulant responses induced by waterpipe smoke exposure in mice

**DOI:** 10.3389/ftox.2026.1696863

**Published:** 2026-02-17

**Authors:** Sumaya Beegam, Nur. E. Zaaba, Ozaz Elzaki, Javed Yasin, Badreldin H. Ali, Abderrahim Nemmar

**Affiliations:** 1 Department of Physiology, College of Medicine and Health Sciences, United Arab Emirates University, Al Ain, United Arab Emirates; 2 Department of Internal Medicine, College of Medicine and Health Sciences, United Arab Emirates University, Al Ain, United Arab Emirates; 3 Independent Researcher, Lincoln, NE, United States

**Keywords:** coagulation, L-2-oxothiazolidine-4-carboxylic acid, nose-only exposure, oxidative stress, thrombosis, waterpipe smoke

## Abstract

**Introduction:**

Exposure to waterpipe smoke (WPS) in humans and experimental animals has been reported to cause oxidative stress and thrombotic complications. L-2-Oxothiazolidine-4-carboxylic acid (OTC) is a cysteine prodrug that maintains glutathione (GSH) in tissues. Nevertheless, the possible mitigating effects of OTC on platelet aggregation induced by WPS inhalation, and its underlying mechanisms of action remain unexplored. This is the goal of the present work in BALB/c mice.

**Methods:**

Animals were exposed to either WPS or air (control) by inhalation daily for 30 min for 1 month. OTC was given 1 h before each exposure session by gavage at a dose of 80 mg/kg.

**Results:**

WPS inhalation increased various markers of platelet aggregation, coagulation, fibrinolysis and endothelial integrity (platelet factor 4, tissue factor, fibrinogen, thrombin-antithrombin complexes, plasminogen activator inhibitor, P-selectin, E-selectin, intercellular adhesion molecule 1 and vascular cell adhesion molecule 1). It also shortened the prothrombin time and partial thromboplastin time and augmented the plasma concentrations of C-reactive protein and triglycerides. All these effects were attenuated by OTC treatment. Likewise, OTC administration significantly mitigated platelet aggregation *in vivo*. Platelets isolated from mice exposed to WPS showed high levels of markers of oxidative and nitrosative stress, calcium, annexin V and calpain. The latter effects were significantly alleviated by OTC treatment.

**Discussion:**

Our data show that OTC administration significantly mitigated WPS-induced *in vivo* endothelial injury and thrombotic events, as well as platelet oxidative stress and apoptosis. This finding provides evidence on the mechanisms of toxicity of WPS on platelet physiology, and the alleviative action of OTC.

## Introduction

Waterpipe smoking (WPS) has tremendously increased, not only in Eastern but also in Western countries in Europe and North America, and is becoming an alarming public health threat and a major worldwide tobacco epidemic (Chaieb and Ben, 2021; Jawad et al., 2018; Nzeako et al., 2025; Sepidarkish et al., 2025). The general public judgement misapprehends the toxicity of WPS and considers it safer as compared with cigarette smoking (Chaieb and Ben, 2021; Jawad et al., 2018). Factors that have augmented the acceptance and use of WPS include fallacies such as that the smoke generated by charcoal-heated WPS is detoxified as it moves via the water, and that WPS is not as addictive as cigarette smoke. These factors also include the use of flavoured fruit aroma waterpipe tobacco, as well as the encouragement and promotion by social media of this mode of tobacco usage (Chaieb and Ben, 2021; Jawad et al., 2018; Nzeako et al., 2025; Sepidarkish et al., 2025). Furthermore, it has been recently reported that WPS is associated with more than doubling of the likelihood of later initiation of cigarette smoking, confirming and supporting the notion that WPS is a gateway for cigarette smoking (Al Oweini et al., 2019).

The cardiovascular effects of WPS have been well-documented in both clinical and laboratory investigations (Chaieb and Ben, 2021; Nzeako et al., 2025; Bhatnagar et al., 2019; Rababa’h et al., 2021; Qasim et al., 2019). It has been reported that WPS triggers an augmentation of plasma nicotine levels, carbon monoxide, myocardial O_2_ demand, blood pressure, lipidaemia, and glycemia (Chaieb and Ben, 2021; Bhatnagar et al., 2019; Rababa’h et al., 2021; Qasim et al., 2019). Also, more burden of atherosclerotic disease and tendency for ST-segment–elevation myocardial infarction have been seen in consumers of waterpipe smoke (Chaieb and Ben, 2021; Bhatnagar et al., 2019; Rababa’h et al., 2021; Qasim et al., 2019). Additionally, accumulating evidence have demonstrated that WPS triggers vascular endothelial injury, platelet activation and coagulation, and that oxidative stress is a key player in these effects (Qasim et al., 2019; Muddathir et al., 2018; Nemmar et al., 2020; Alar et al., 2022; Badran and Laher, 2020; Hamadi et al., 2024; Nemmar et al., 2023).

Although the toxicological and cardiovascular mechanisms of WPS have been extensively characterized—implicating oxidative stress, endothelial dysfunction, inflammation, nitric oxide depletion, and platelet hyperreactivity as major contributors to vascular injury (Sepidarkish et al., 2025; Rababa’h et al., 2021; Alar et al., 2022; Badran and Laher, 2020; Alarabi et al., 2020; Nemmar et al., 2019; Nemmar et al., 2017a)—there remains a need for interventions that directly counteract these oxidative and thrombotic pathways. L-2-oxothiazolidine-4-carboxylic acid (OTC; procysteine) is a cysteine prodrug that is intracellularly converted by 5-oxoprolinase into cysteine, the rate-limiting precursor for glutathione (GSH) synthesis (Angelovski et al., 2023; Angelovski et al., 2022; Averill-Bates, , 2023). GSH is the principal intracellular antioxidant that protects endothelial cells and platelets from oxidative injury (Averill-Bates, , 2023). Compared with NAC, foundational hepatocyte studies demonstrated more efficient intracellular cysteine delivery and GSH replenishment with OTC, while *in vivo* investigations have shown that OTC effectively restores hepatic and cellular GSH levels, improves redox homeostasis, and confers vascular protection in oxidative stress models (Banks and Stipanuk, 1994; Deevska et al., 2014; Aldini et al., 2018; Giustarini et al., 2023; Lee et al., 2005; Patel et al., 2021). OTC administration has also been reported to re-establish GSH stores and reduce brain infarct injury in a mouse stroke model, to mitigate liver injury and fibrosis through modulation of nuclear factor erythroid 2-related factor 2 in rats, and to attenuate airway hyperreactivity in a mouse model of asthma (Liu et al., 2020; Lee et al., 2006; Kim et al., 2012). In addition, we have previously demonstrated that OTC exerts protective actions against acute (24 h) intratracheal instillation of diesel exhaust particle-induced pulmonary and systemic inflammation and pial venule thrombosis in mice (Nemmar et al., 2009).

Accordingly, in this study, OTC was employed as an experimental tool to delineate the role of GSH-dependent redox modulation in WPS-induced oxidative vascular injury, while acknowledging its current non-approved status and the need for further preclinical and clinical evaluation. Hence, we hypothesized that restoration of intracellular GSH levels by OTC would mitigate endothelial injury, platelet activation, and coagulation abnormalities elicited by sub-chronic WPS exposure. These interactions have not been previously investigated. Consequently, the present study aimed to (1) assess the potential alleviating effects of OTC on sub-chronic (1 month) WPS exposure-induced endothelial injury, coagulation, and platelet aggregation *in vivo*, and (2) elucidate the mechanisms underlying these effects by examining platelet physiological responses, including oxidative stress and apoptosis.

## Materials and methods

### Animals and treatments

This project was evaluated and endorsed by the United Arab Emirates University Review Board of the United Arab Emirates University, and experiments were performed in agreement with protocols accepted by the Institutional Animal Ethics Committee #ERA_20195983.

### WPS exposure

BALB/c mice (Taconic Farms Inc., Germantown, NY, United States) were kept in a conventional animal house and sustained on a 12 h light-dark cycle (lights switched on at 6a.m.). The mice were kept in cages and provided *ad libitum* with pelleted food and water. After 5 days of adjustment, mice were randomly segregated into air-exposed (control) and WPS-exposed groups.

Animals were placed in soft restraints which were connected to the WPS exposure tower (Nemmar et al., 2013a). Mice were exposed to either WPS or air by inhalation via their noses with a nose-only exposure setup coupled to a waterpipe device (InExpose System, Scireq, Canada). Mice were exposed to an apple-flavoured tobacco which is available commercially and consisting of tobacco, molasses, glycerin and natural flavor with nicotine (0.5%) (Al Fakher Tobacco Trading, UAE). Tobacco was set alight with an instant light charcoal disk. Similar to WPS consumption in humans, the smoke from the waterpipe goes through the water before it was aspirated into the WPS exposure tower. A computer-based system was used to monitor exposure procedure (InExpose System, Scireq, Canada). A computer-monitored puff was produced every min resulting in a WPS puff duration of 2s followed by fresh air exposure of 58s. The length of an exposure session was 30 min/day. Control mice were exposed air-only using the same protocol. The WPS exposure session protocol used here is similar to those reported by experimental animal and human studies (Nemmar et al., 2013a; Toukan et al., 2020; Hakim et al., 2011). Mice were exposed daily for a duration of a month. One hour before the exposure to either WPS or air, OTC (80 mg/kg) (Sigma-Aldrich Co., St Louis, MO, United States) dissolved in distilled water, or distilled water was administered by gavage to mice. The selected dose of OTC used in the present study was chosen based on previously published *in vivo* studies demonstrating effective antioxidant and anti-inflammatory protection at comparable doses in mice (Nemmar et al., 2009; Lee et al., 2004; Choi et al., 2013).

### Blood samples collection and analysis

Following the exposure period to either air or WPS with or without OTC treatment, mice were anesthetized by intraperitoneal injection of sodium pentobarbital (60 mg/kg), and then blood was collected from the inferior vena cava in EDTA (4%) and centrifuged at 4 °C for 15 min at 900 *g*, and the plasma samples collected were kept at −80 °C pending analysis.

### Measurement of C-reactive protein (CRP) fibrinogen, platelet factor 4 (PF4), tissue factor, thrombin–antithrombin (TAT) complexes, plasminogen activator inhibitor-1 (PAI-1), P-selectin, E-selectin, intercellular adhesion molecule 1 (ICAM-1) and vascular cell adhesion molecule 1 (VCAM-1) and lipid profile concentrations in plasma

The plasma concentrations of CRP (DY1829), PF4 (DY595), PAI-1 (DY3828), P-selectin (DY737), E-selectin, ICAM-1 (DY796), and VCAM-1 (DY643) were measured using commercially available ELISA kits from R&D Systems (DuoSet; Minneapolis, MN, United States). Fibrinogen was measured using an ELISA kit from Aviva Systems Biology (OKIA00093; San Diego, CA, United States). Plasma TAT complex levels were quantified using a mouse ELISA kit from MyBioSource (MBS760664; San Diego, CA, United States). HDL, LDL, and triglycerides were analyzed using COBAS INTEGRA 400 Plus/E411 from Roche Diagnostics (Indianapolis, IN, United States).

### Mouse pial microvessel thrombosis model

In separate sets of mice, *in vivo* pial arteriolar and venular thrombogenesis was evaluated after the exposure to either WPS or air, with or without OTC treatment, as per a previously reported technique (Nemmar et al., 2013a; Nemmar et al., 2017b). In brief, the experimental procedure began with anesthesia induced by intraperitoneal injection of urethane (1 mg/g body weight). Following this, the trachea was intubated to secure the airway, and a 2F venous catheter (Portex, Hythe, United Kingdom) was inserted into the right jugular vein to permit intravenous administration of fluorescein (Sigma-Aldrich, St. Louis, MO, United States). A craniotomy was then carried out over the right temporoparietal cortex using a hand-held microdrill, after which the dura mater was carefully removed. Preparations exhibiting any visible damage to cerebral microvessels or underlying brain tissue were excluded from further analysis. Cerebral microcirculation was visualized in real time using a fluorescence microscope (Olympus, Melville, NY, United States) equipped with a digital camera and DVD recording system. Throughout the experiment, the animal’s body temperature was maintained at 37 °C with a heating pad and continuously monitored via a rectal thermoprobe connected to a temperature monitor (Physitemp Instruments, NJ, United States). A microscopic field containing arterioles and venules (15–20 µm in diameter) was selected for observation and recorded before and during photochemical stimulation. For the induction of photochemical injury, fluorescein (0.1 mL of a 5% solution per mouse) was administered through the jugular catheter and allowed to circulate for 30–40 s. The cranial preparation was then illuminated with stabilized mercury light, initiating localized endothelial damage. This photochemically induced vascular injury triggered platelet adhesion and aggregation at the affected sites, leading to progressive thrombus formation and, ultimately, complete vascular occlusion. The latency from the onset of illumination to full cessation of blood flow (“thrombotic occlusion time”) was measured in seconds for both arterioles and venules. Upon completion of all recordings, animals were euthanized with an overdose of urethane to ensure humane termination of the experiment.

### Prothrombin time (PT) and activated partial thromboplastin time (aPTT) measurement in plasma *in vitro*

Following the exposure to either WPS or air with or without OTC treatment, animals were anesthetized, blood was collected from the inferior vena cava and kept in citrate solution (3.2%) (proportion of blood to anticoagulant: 9:1). The PT was assessed on freshly obtained platelet-poor plasma with human relipidated recombinant thromboplastin (Recombiplastin; Instrumentation Laboratory, Orangeburg, NY, United States) and the aPTT was determined with the automated aPTT reagent from bioMerieux (Durham, NC, United States) using a coagulometer (MC 1 VET, Merlin, Lemgo, Germany) (Nemmar et al., 2019; Nemmar et al., 2013a).

### Preparation of platelets and platelet-related biochemical analysis

Following the exposure to either WPS or air with or without OTC treatment, washed platelets were prepared from collected blood as reported earlier (Towhid et al., 2013; Nemmar et al., 2015). The blood was spun for 20 min at 200 g at 25 °C. The platelet-rich plasma was separated, supplemented with Tyrode buffer (137 mM NaCl, 2.8 mM KCl, 0.4 mM Na_2_HPO_4_, 12 mM NaHCO_3_, 5 mM glucose, 10 mM HEPES, 0.1% BSA), pH 6.5 in 1:7 volumetric ratio and spun for 10 min at 900 g. The platelet pellet was resuspended in 1 mL of Tyrode buffer (pH 7.4), standardized to a concentration of 1 million platelets in a total volume of 1 mL. The platelet aliquots were added into homogenizer tubes with grinding beads (Myung et al., 2017; Myung et al., 2020). Thereafter, the platelets were treated with a bead mill homogenizer (Precellys 24, OMNI International) with three cycles at 6000 rpm for 20s as described before (Myung et al., 2017; Myung et al., 2020). The disrupted platelet homogenate was centrifuged at room temperature for 20 min at 12,000 g to precipitate the cellular debris. The collected supernatants were filtered (0.2-μm filter) and the obtained platelet releasates were kept at −80 °C until further analysis.

Markers of oxidative stress were measured in the obtained supernatants. The levels of NADPH-dependent membrane lipid peroxidation were quantified as thiobarbituric acid reactive substances using malondialdehyde as a standard, following the TBARS assay protocol (Cayman Chemical, 10009055; Ann Arbor, MI, United States) (Nemmar et al., 2013b; Ali et al., 2018). Reactive oxygen species (ROS) were quantified using 2′,7′-dichlorofluorescein diacetate (DCFDA; Sigma–Aldrich Fine Chemicals, D6883; St. Louis, MO, United States) as a fluorescent indicator, following previously established protocols (LeBel et al., 1990; Hamadi et al., 2025). The determination of GSH concentrations was performed using a commercially available kit (Sigma–Aldrich Fine Chemicals, CS0260; Munich, Germany). Measurement of catalase activity was carried out using a spectrophotometric method with a commercially available kit from Cayman Chemical (707002; Ann Arbor, MI, United States). Nitric oxide (NO) levels were measured using a total NO assay kit from Cayman Chemical (780001; Ann Arbor, MI, United States), which quantifies the stable NO metabolites NO_2_^−^ and NO_3_^−^.

In separate experiments, washed platelets, prepared as described above, were used to quantify platelet intracellular Ca^2+^ levels using the fluorescent indicator dye Fura-2a.m. (Calbiochem; La Jolla, CA, United States), according to a modified version of previously described techniques (Nemmar et al., 2015; Vicari et al., 1994; Ferdous et al., 2018; Pignatelli et al., 2003; Grynkiewicz et al., 1985). Briefly, platelets were incubated with 5 µL of Fura-2a.m. for 15 min at 37 °C in the dark to allow cellular uptake and enzymatic cleavage of the acetoxymethyl ester to the fluorescent Fura-2 form. The loaded platelets were then washed, resuspended in Ringer’s solution, and further incubated for 30 min under the same conditions. Finally, Ca^2+^-dependent fluorescence intensity was monitored with a fluorometer (SFM 25, Kontron; Zurich, Switzerland) set at 340 nm excitation and 510 nm emission. Measurements represent total fluorescence intensity and were expressed as relative fluorescence units (RFU) for comparison among the Air, WPS, OTC + Air, and OTC + WPS groups.

Annexin V was quantified in platelet lysates using a mouse Annexin V (ANXA5) ELISA kit (Elabscience, Texas, United States) according to the manufacturer’s instructions. This assay detects total Annexin V protein—including both membrane-associated and cytosolic fractions—rather than surface-bound Annexin V alone. Briefly, platelet lysates were added to wells pre-coated with anti-Annexin V antibodies, followed by incubation with a biotinylated detection antibody and horseradish peroxidase (HRP)-streptavidin conjugate. After addition of the substrate solution, absorbance was measured at 450 nm using a microplate reader. The results were expressed as optical density (OD) values proportional to the total Annexin V content in platelet samples.

The activated calpain was fluorometrically quantified by means of a calpain activity assay kit as per the vendor’s instruction (Genway Biotech, San Diego, United States).

### Statistics

All analysis and graphs were produced using GraphPad Prism Version 7 for Windows software (Graphpad Software Inc., San Diego, United States). We have applied one-way analysis of variance (ANOVA) followed by multiple comparisons using Holm-Sidak’s method. Data were expressed as means ± SEM. *P* values less than 0.05 were considered significant.

## Results

### Impact of WPS on plasma concentrations of markers of coagulation, and the effect of OTC treatment thereon

**FIGURE 1:**
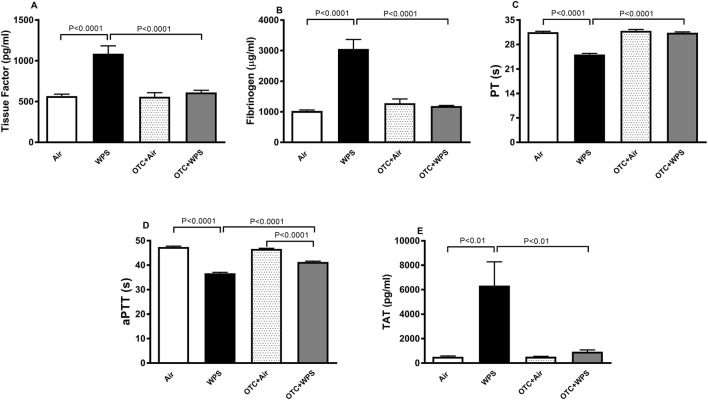
Plasma tissue factor **(A)**, plasma fibrinogen **(B)**, prothrombin time (PT, **(C)**, activated partial thromboplastin time [aPTT, **(D)**], and plasma thrombin–antithrombin (TAT) complexes **(E)** at the end of the 1-month exposure period to air (control) or waterpipe smoke (WPS), with or without L-2-oxothiazolidine-4-carboxylic acid (OTC) treatment. Data are mean ± SEM (n = 8).

Figure 1 illustrates the effect of the exposure of either air or WPS, with or without OTC treatment on blood markers of coagulation. Compared with control mice, WPS inhalation for 1 month triggered a statistically significant (P < 0.0001) increase of plasma concentrations of tissue factor and fibrinogen (Figures 1A,B). The latter effects were significantly (P < 0.0001) mitigated by OTC treatment. Compared with the control group, WPS exposure induced a significant (P < 0.0001) shortening of aPTT and PT (Figures 1C,D). These effects were significantly alleviated by the treatment with OTC (P < 0.0001). The aPTT in OTC + WPS group was significantly (P < 0.0001) shorter compared with OTC + air group. Plasma levels of TAT complexes were significantly (P < 0.01) increased in mice exposed to WPS compared with the control group, indicating enhanced thrombin generation and procoagulant activity. Treatment with OTC markedly (P < 0.01) reduced TAT concentrations toward control values (Figure 1E). These results confirm that WPS exposure promotes systemic coagulation activation, which is effectively mitigated by OTC administration.

### Effect of WPS on plasma concentrations of PF4 and PAI-1 and the influence of OTC treatment thereon

**FIGURE 2:**
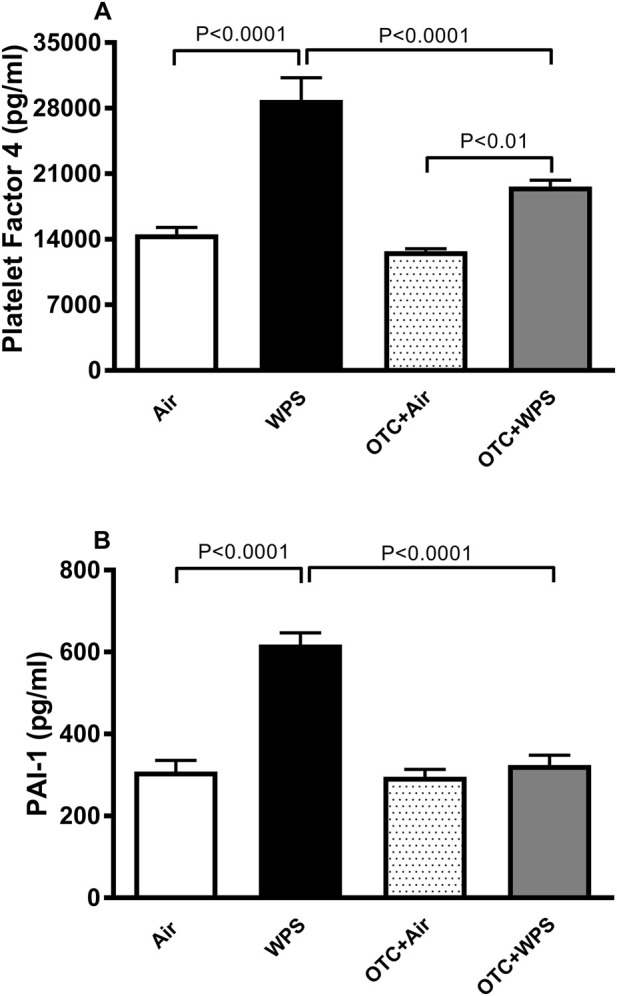
Platelet factor 4 **(A)** and plasminogen activator inhibitor-1 [PAI-1, **(B)**] concentrations in the plasma at the end of 1-month exposure period to air (control) or waterpipe smoke (WPS), with or without L-2-oxothiazolidine-4-carboxylic acid (OTC) treatment. Data are mean ± SEM (n = 8).

Figure 2 displays the effects inhalation of either WPS or air on the plasma concentration PF4, a maker of platelet activation (degranulation), and PAI-1 which is derived primarily from the vessel wall. WPS inhalation induced a significant (P < 0.0001) increase of PF4 and PAI-1 concentrations in plasma (P < 0.0001). These effects were significantly alleviated following the administration of OTC (P < 0.0001).

### Effect of WPS on plasma concentrations of P-selectin, E-selectin, VCAM-1 and ICAM-1 and the influence of OTC treatment thereon

**FIGURE 3:**
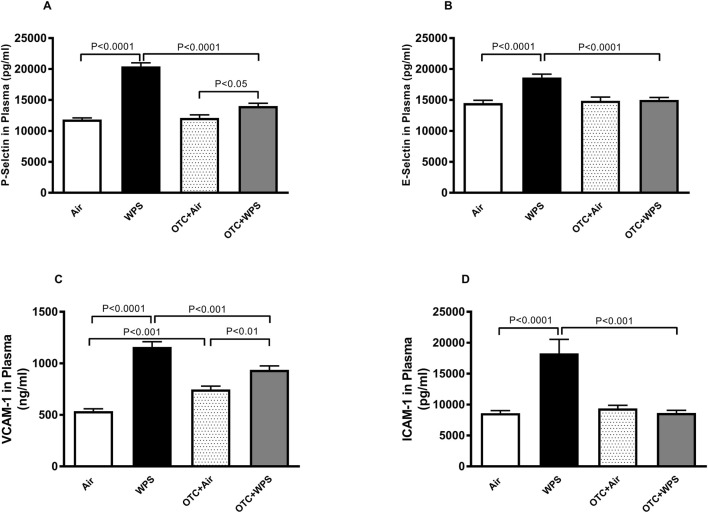
Plasma P-selectin **(A)**, E-selectin **(B)**, vascular cell adhesion molecule 1 [VCAM-1, **(C)**] and intercellular adhesion molecule 1 [ICAM-1, **(D)**] at the end of at the end of 1-month exposure period to air (control) or waterpipe smoke (WPS), with or without L-2-oxothiazolidine-4-carboxylic acid (OTC) treatment. Data are mean ± SEM (n = 8).

Figure 3 depicts the impact of the exposure of either WPS or air inhalation on the plasma concentrations of P-selectin, E-selectin, VCAM-1 and ICAM-1. After the exposure to WPS, the concentrations of P-selectin, E-selectin, VCAM-1 and ICAM-1 in the plasma were significantly (P < 0.0001) augmented compared with the air-exposed group. OTC administration induced a significant (P < 0.0001-P<0.001) alleviation of these observed effects.

### Impact of WPS on plasma concentrations of CRP, triglyceride, LDL and HDL, and the effect of OTC treatment

**FIGURE 4:**
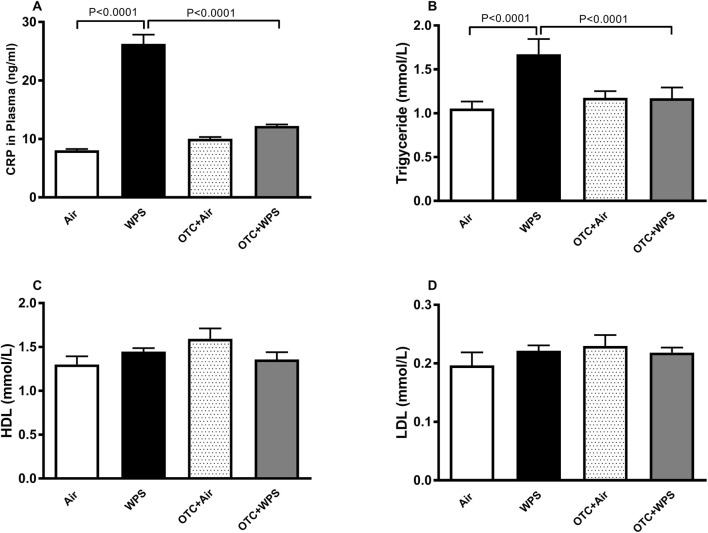
Plasma C-reactive protein [CRP, **(A)**], triglyceride **(B)**, high-density lipoprotein [HDL, **(C)**] and low-density lipoprotein [LDL, **(D)**] at the end of the 1-month exposure period to air (control) or waterpipe smoke (WPS) with or without L-2-oxothiazolidine-4-carboxylic acid (OTC) treatment. Data are mean ± SEM (n = 5–6).

WPS exposure induced a significant increase in CRP (P < 0.0001), and the OTC treatment completely prevented this effect (Figure 4A). The lipid profile of mice exposed to either air or WPS with or without OTC treatment is shown in Figures 4B–D. Compared with control group, WPS inhalation induced a significant increase in triglyceride concentrations (P < 0.0001). This effect was completely prevented following OTC treatment. However, WPS exposure did not alter the plasma concentrations of LDL or HDL.

### Effect of WPS on thrombotic occlusion time in photochemically-injured pial arterioles and venules and the influence of OTC treatment thereon

**FIGURE 5:**
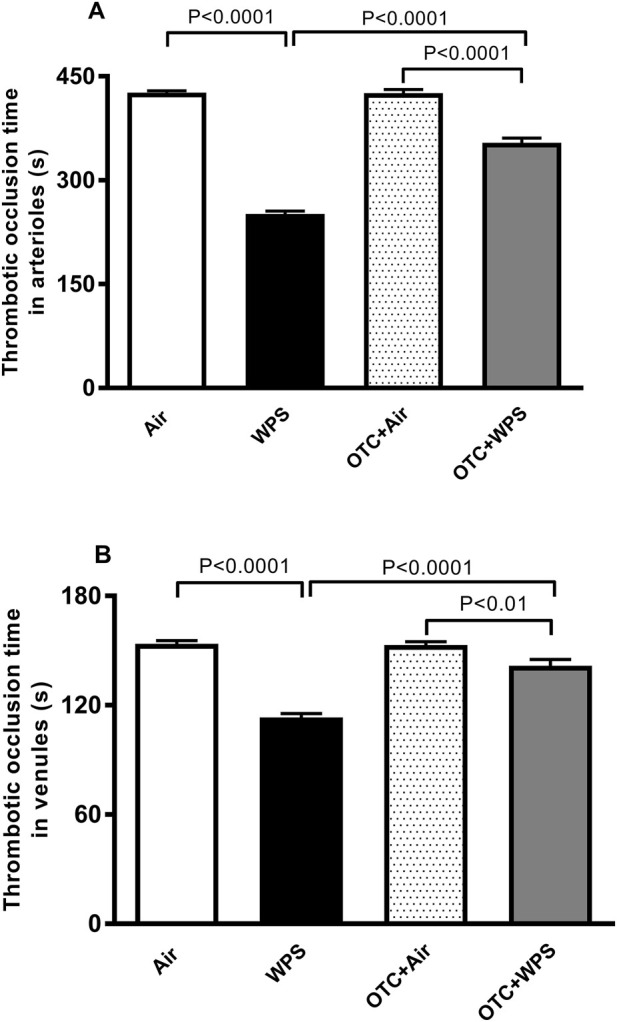
Thrombotic occlusion time in pial arterioles **(A)** and venules **(B)** at the end of 1-month exposure period to air (control) or waterpipe smoke (WPS) with or without L-2-oxothiazolidine-4-carboxylic acid (OTC) treatment. Data are mean ± SEM (n = 8).

Compared with air-exposed mice, WPS inhalation for 1 month triggered a significant reduction of the thrombotic occlusion time in pial arterioles and venules of mice (P < 0.0001) (Figures 5A,B). The reduction of the thrombotic occlusion time in arterioles and venules induced by WPS was significantly mitigated by OTC treatment (P < 0.0001) (Figure 5). The thrombotic occlusion times in pial arterioles (P < 0.0001) and venules (P < 0.01) were significantly shorter in OTC + WPS group versus the OTC + air group.

### Impact of WPS on platelet levels of LPO, ROS, GSH, catalase and NO, and effect of OTC treatment

**FIGURE 6:**
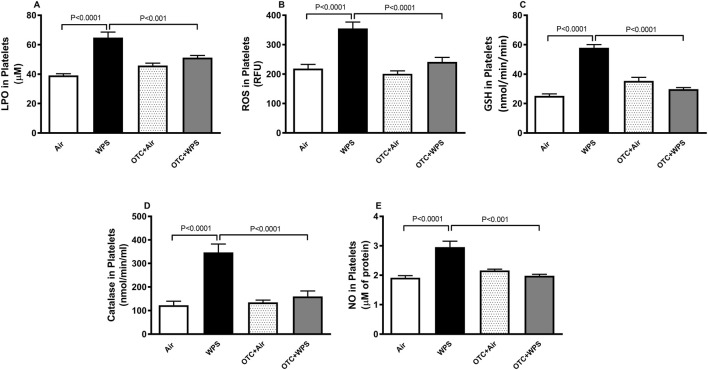
Platelet levels of lipid peroxidation [LPO, **(A)**], reactive oxygen species [ROS, **(B)**], glutathione [GSH, **(C)**], catalase **(D)**, and total nitric oxide [NO, **(E)**] at the end of the 1-month exposure period to air (control) or waterpipe smoke (WPS) with or without L-2-oxothiazolidine-4-carboxylic acid (OTC) treatment. Data are mean ± SEM (n = 7–8).

The effects of exposure to either air or WPS, with or without OTC treatment, on platelet oxidative stress markers are shown in Figure 6. Compared with control mice, sub-chronic (1 month) WPS inhalation induced a significant (P < 0.0001) increase in platelet levels of LPO, ROS, GSH, catalase, and NO. These elevations in LPO and ROS confirm the induction of oxidative stress, whereas the concurrent increases in GSH and catalase likely reflect a compensatory antioxidant response to counteract the oxidative challenge. NO, a free radical scavenger, also increased following WPS exposure, consistent with an adaptive response to oxidative imbalance. Treatment with OTC significantly attenuated all these changes in the OTC + WPS group compared with WPS alone (P < 0.0001-P < 0.001), suggesting that OTC mitigated both the oxidative load and the secondary antioxidant response (Figures 6A–E).

### Impact of WPS on platelet levels of calcium, annexin V and calpain and the effect of OTC treatment

**FIGURE 7:**
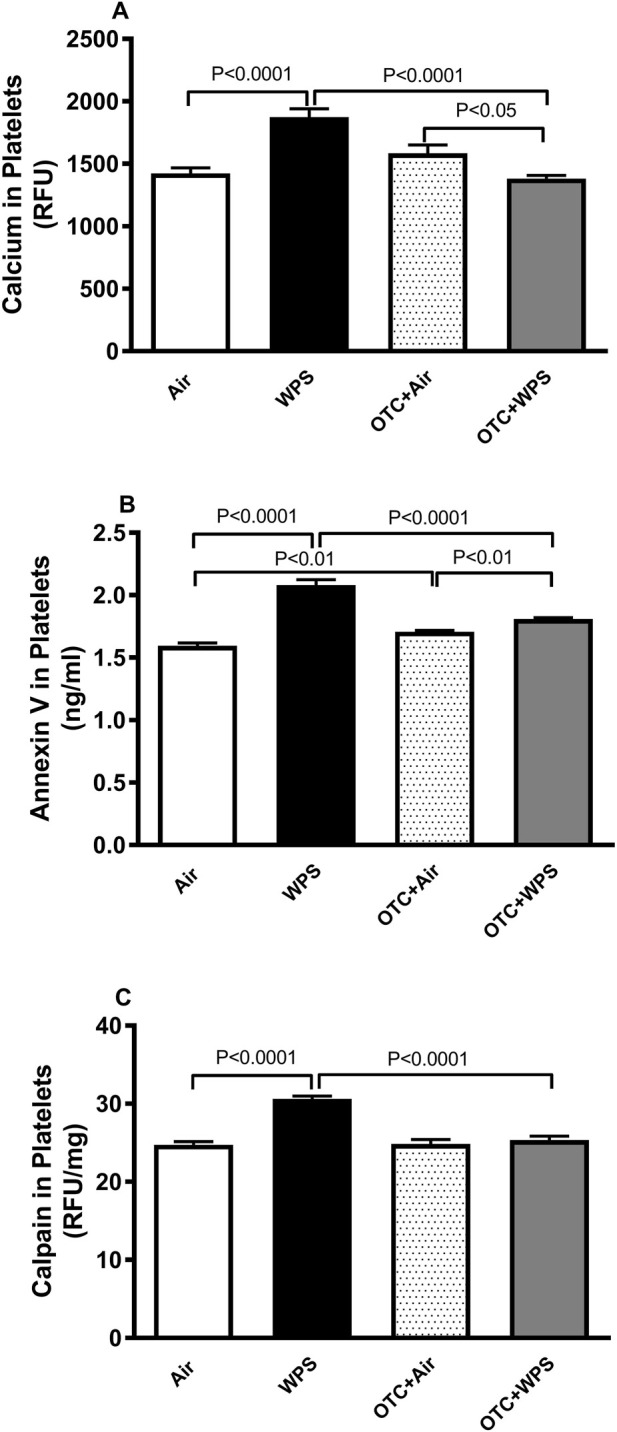
Calcium **(A)**, annexin V **(B)** and calpain **(C)** levels in platelets at the end of at the end of 1-month exposure period to air (control) or waterpipe smoke (WPS), with or without L-2-oxothiazolidine-4-carboxylic acid (OTC) treatment. Data are mean ± SEM (n = 8).

WPS inhalation for 1 month induced a significant (P < 0.0001) increase in the platelet levels of intracellular calcium, annexin V and calpain (Figure 7). Compared with WPS group, mice exposed to WPS and treated with OTC displayed a significant reduction in the levels of calcium, annexin V and calpain (P < 0.0001) (Figure 7). There were slight but statistically significant (P < 0.01-P<0.05) differences in the levels of calcium and annexin V between OTC + air and OTC + WPS groups.

## Discussion

The present study provides experimental evidence that OTC administration significantly mitigates WPS-induced *in vivo* endothelial injury and thrombotic events, as well as platelet oxidative stress and apoptotic changes.

Numerous epidemiological and clinical studies have highlighted that an increased intake of antioxidants is accompanied with a positive impact on several systems comprising the circulatory system (Siti et al., 2015; Baradaran et al., 2014). GSH is the most vital antioxidant produced intracellularly. It plays as a key role in the mechanism of antioxidant defence to counterbalance oxidative stress by directly scavenging reactive oxygen and nitrogen species and exerts an essential role in various metabolic and cellular processes by preserving redox homeostasis (Wrotek et al., 2020; Marí et al., 2020). When GSH clears reactive oxygen species, it is transformed to oxidized GSH or glutathione-disulfide. The depletion of GSH alters mitochondrial ATP generation and triggers cell death signaling pathways (Marí et al., 2020). Deficiency of GSH increases the cellular vulnerability for oxidative damage, and consequently, GSH imbalance is seen in several and varied pathophysiological conditions including cardiovascular, respiratory, metabolic and neurodegenerative diseases (Siti et al., 2015; Baradaran et al., 2014; Wrotek et al., 2020; Marí et al., 2020). OTC has been reported to exert an effective antioxidant effect in various animal models including ischemic stroke, lung injury, retinal pigment epithelial cells damage, dialysate-induced senescence in human peritoneal mesothelial cells and efferocytosis in the airway of a smoking murine model (Lee et al., 2005; Liu et al., 2020; Nemmar et al., 2009; Promsote et al., 2014; Sosinska-Zawierucha et al., 2018; Hodge et al., 2011). While it well established that oxidative stress plays a critical role in WPS-induced pathophysiological effects in various systems including the circulatory system, the possible positive impact of OTC thereon has, as far as we are aware, not been studied so far. The dose of OTC employed in the present work (80 mg/kg) was selected based on previously published studies (Kim et al., 2012; Nemmar et al., 2009).

Our findings show that WPS inhalation caused a substantial increase in tissue factor and fibrinogen concentrations in plasma and that OTC treatment significantly prevented this effects. Various studies have reported that exposure to cigarette smoke or WPS causes a significant increase in the concentration of fibrinogen and tissue factor (Nemmar et al., 2019; Delgado et al., 2015; Nemmar et al., 2017c; Caponnetto et al., 2011). Fibrinogen is a well-known acute-phase protein that augments blood viscosity and enhances thrombogenicity and tissue factor which can be released from both activated circulating monocytes and from vascular wall is responsible for the initiation of the extrinsic pathway of blood coagulation cascade (Delgado et al., 2015; Del et al., 2015). Moreover, our data demonstrate that WPS exposure caused a significant reduction of PT and aPTT. OTC treatment normalized the shortening of PT induced by WPS, which is in complete agreement with the finding that fibrinogen also returned back to control level in OTC + WPS group. OTC normalized the shortening of aPTT triggered by WPS inhalation only partially. This indicate that there is a small effect remaining that reflects other aspects of intrinsic coagulation besides fibrinogen, that are not completely prevented. Thus, WPS may have a direct effect on factor VIII, IX, independently of the effects normalized by OTC. In addition to these findings, plasma TAT complexes were significantly increased following WPS exposure, indicating increased thrombin generation and systemic procoagulant activity. This observation aligns with the elevated tissue factor and fibrinogen concentrations and the shortened PT and aPTT, further supporting that WPS exposure promotes coagulation activation. Importantly, OTC treatment markedly reduced TAT levels toward control values, indicating effective attenuation of WPS-induced thrombin generation. Similar increases in TAT complexes have been reported in smokers and in models of oxidative stress–induced thrombosis, reflecting heightened thrombin formation and endothelial activation (Caponnetto et al., 2011; Ambrose and Barua, 2004; Gallucci et al., 2020; Hioki et al., 2001).

PF4 is a positively charged tetramer belonging to the CXC chemokine family, which is a marker of platelet activation (degranulation) and PAI-1 is derived primarily from the vessel wall and is the utmost effective endogenic inhibitor of fibrinolysis and is involved in the pathogenesis of several cardiovascular diseases (Nevzorova et al., 2019; Cesa et al., 2010). Our data show that OTC treatment induced a substantial alleviation of WPS-induced increase in the plasma concentration of PF4 and PAI-1.

Furthermore, our data show that exposure to WPS for 1 month was accompanied with a significant elevation of plasma concentrations of soluble P-selectin, E-selectin, VCAM-1 and ICAM-1, and that these effects were significantly alleviated in mice exposed to WPS and treated with OTC. P-selectin, E-selectin, VCAM-1 and ICAM-1 are cell adhesion molecules and surrogate markers of endothelial function (Qiu et al., 2019; Theofilis et al., 2021). Also, it is well-known that P-selectin is released both from activated platelets and from activated endothelial cells (from the membrane and Weibel-Palade bodies) (Qiu et al., 2019; Theofilis et al., 2021). The VCAM-1 and ICAM-1 elevation confirms the endothelial cell damage, which is turn is prothrombotic by inducing the activation of both platelet and coagulation (Qiu et al., 2019; Theofilis et al., 2021). Several clinical studies have reported that increased serum concentrations of these cell adhesion molecules may be independent risk factors for atherosclerosis, cardiovascular disease, diabetes and smoking (Qiu et al., 2019; Theofilis et al., 2021; Khan et al., 2020; Demerath et al., 2001).

A significant increase in the concentrations of LDL and triglyceride has been reported in waterpipe smokers (Saffar et al., 2018). A recent experimental study found that whole body exposure to WPS in male rats for 19 weeks led to a statistically insignificant increase in the concentrations of triglyceride and LDL (Al-Sawalha et al., 2020). In the present study, we used mice of both gender and exposed them using the nose-only exposure system for 1 month and found a significant increase in triglyceride concentration. The disparity between our study and the former one could be ascribed to the animal species used, gender, exposure system, the exposure regimen, or to other unknow factors. Interestingly, our data show that the treatment with OTC prevented the increase of triglyceride induced by WPS. It has been reported that exposure to cigarette smoke induce an increase in lipid profile including triglyceride and that the treatment with the antioxidant (−)-epigallocatechin-gallate reversed this effect (Gokulakrisnan et al., 2011). The increase of triglyceride seen here could also be ascribed to acute phase response in the liver in response to WPS inhalation. This is in agreement with the CRP and fibrinogen data, produced in the liver.

We have previously reported that subchronic and chronic exposure to WPS aggravate thrombosis in pial microvessels (Nemmar et al., 2017a; Nemmar et al., 2013a). Moreover, we have previously reported that OTC administration prevented thrombosis in cerebral microvessels of mice acutely (24 h) exposed to diesel exhaust particles by intratracheal instillation (Nemmar et al., 2009). However, the effects and the underlying mechanisms of OTC on subchronic WPS inhalation-induced thrombotic events has not been investigated before. The present data revealed that WPS exposure caused an exacerbation of thrombosis in the pial arterioles and venules. This is in agreement with vascular damage. In addition, because the effect is as pronounced in the venules as in the arterioles, this points to circulating platelet activation which is in agreement with the elevated plasma concentration of PF4. Our data revealed that OTC treatment significantly alleviated these effects.

Our *in vivo* model of thrombosis in pial arterioles and venules relies on platelet activation and aggregation (al Dhaheri et al., 1995). Using this model, 1 month of WPS inhalation significantly accelerated thrombus formation, as evidenced by shortened thrombotic occlusion times in both arterioles and venules. OTC treatment significantly attenuated the WPS-induced reduction in occlusion time.

Since we showed that OTC exerts a protective effect against WPS inhalation-induced platelet aggregation *in vivo*, and to gain more insight into the mechanisms of action of OTC, we have evaluated, *ex vivo*, several markers of oxidative stress in platelets isolated from mice exposed for 1 month to WPS or air and treated with OTC. Our data show that levels of LPO, ROS, and the free radical scavenger NO were significantly increased in platelets from the WPS-exposed group, accompanied by elevated concentrations of the endogenous antioxidants GSH and catalase. This pattern reflects an oxidative challenge with a concurrent compensatory antioxidant response, indicating that WPS exposure induces oxidative stress. Notably, co-administration of OTC with WPS markedly attenuated these changes, underscoring the capacity of OTC to counteract WPS-induced oxidative and nitrosative imbalance through its GSH-dependent antioxidant action. It is well established that resting platelets keep a low cytosolic calcium concentration and a steep plasma membrane calcium gradient (Varga-Szabo et al., 2009; Masselli et al., 2020). Nevertheless, upon activation, a substantial elevation of cytosolic calcium is seen that contributes to several phases of cellular activation, including reorganization of the actin cytoskeleton, degranulation and aggregation (Varga-Szabo et al., 2009; Masselli et al., 2020). Along with the occurrence of oxidative stress, our findings show that the exposure to WPS increase the concentrations of intracellular calcium which points to a higher degree of platelet “activatability” towards triggers, and that OTC significantly prevented this effect. It has been previously reported that exposure to platelets to amorphous silica nanoparticles induce oxidative stress and elevation in the intracellular calcium concentrations (Nemmar et al., 2015). Apoptosis or programmed cell death, is crucial for the normal functioning and subsistence of most multi-cellular organisms. It is well-known that oxidative stress plays a key role in apoptosis and antioxidants such as N-acetylcysteine can prevent cells death from apoptosis (Masselli et al., 2020). Annexin V is a calcium dependent phospholipid-binding protein which has an elevated affinity for phosphatidylserine, and binds to cells with exposed phosphatidylserine (Myung et al., 2017). Our data show that exposure to WPS increased total Annexin V expression, and that OTC treatment alleviated this effect. The elevated Annexin V levels may reflect enhanced platelet activation or apoptotic signaling, processes often associated with phosphatidylserine exposure and procoagulant activity at the platelet surface. The observed increase in total Annexin V protein parallels the elevated plasma TAT levels, suggesting enhanced platelet activation and potential phosphatidylserine exposure contributing to thrombin generation, a process mitigated by OTC treatment (Caponnetto et al., 2011; Ambrose and Barua, 2004; Gallucci et al., 2020; Hioki et al., 2001). It has been recently reported that exposure to e-cigarettes or nanoparticles induces platelet aggregation and the expression of annexin V (Nemmar et al., 2015; Qasim et al., 2018). Calpain is a calcium dependent cysteine protease that exists in the form of proenzymes and has been shown to cleave cytoskeletal proteins and induce platelet apoptosis (Nevzorova et al., 2019; Zhang et al., 2011). Our data show that WPS induced a significant increase of calpain which could be ascribed to the elevation in cytosolic calcium triggered by the oxidative stress (Ferdous et al., 2018; Nevzorova et al., 2019; Zhang et al., 2011; Bernhard et al., 2005). The latter effect was completely prevented by OTC treatment, indicating the key role of oxidative stress in the observed effects.

It should be noted that this study has some limitations. Although both male and female mice were used to reduce bias, future studies are needed to assess possible gender-related effects. In addition, Annexin V was measured in platelet lysates to quantify total protein expression rather than surface exposure by flow cytometry. Future work using flow cytometric analysis will help clarify these aspects.

In conclusion, the present data demonstrate that OTC treatment significantly ameliorates WPS-induced *in vivo* thrombotic events and endothelial injury, as well as platelet oxidative stress and apoptotic changes. These findings provide insight into the mechanisms underlying WPS-induced platelet dysfunction and highlight the protective actions of OTC.

## Data Availability

The original contributions presented in the study are included in the article/supplementary material, further inquiries can be directed to the corresponding author.
